# BAFF Index and CXCL13 levels in the cerebrospinal fluid associate respectively with intrathecal IgG synthesis and cortical atrophy in multiple sclerosis at clinical onset

**DOI:** 10.1186/s12974-016-0785-2

**Published:** 2017-01-17

**Authors:** M. Puthenparampil, L. Federle, S. Miante, A. Zito, E. Toffanin, S. Ruggero, M. Ermani, S. Pravato, D. Poggiali, P. Perini, F. Rinaldi, P. Gallo

**Affiliations:** 1 Multiple Sclerosis Centre, Department of Neurosciences DNS, University Hospital–Medical School, via Giustiniani 5, 3518 Padova, Italy; 2 Department of Neurosciences DNS, University Hospital–Medical School, via Giustiniani 5, 3518 Padova, Italy

**Keywords:** Multiple sclerosis, BAFF, CXCL13, IL21, Cerebrospinal fluid, IgGOB, FLS, Intrathecal IgG synthesis

## Abstract

**Background:**

B lymphocytes are thought to play a relevant role in multiple sclerosis (MS) pathology. The in vivo analysis of intrathecally produced B cell-related cytokines may help to clarify the mechanisms of B cell recruitment and immunoglobulin production within the central nervous system (CNS) in MS.

**Methods:**

Paired cerebrospinal fluid (CSF) and serum specimens from 40 clinically isolated syndrome suggestive of MS or early-onset relapsing-remitting MS patients (CIS/eRRMS) and 17 healthy controls (HC) were analyzed for the intrathecal synthesis of IgG (quantitative formulae and IgG oligoclonal bands, IgGOB), CXCL13, BAFF, and IL-21. 3D-FLAIR, 3D-DIR, and 3D-T1 MRI sequences were applied to evaluate white matter (WM) and gray matter (GM) lesions and global cortical thickness (gCTh).

**Results:**

Compared to HC, CIS/eRRMS having IgGOB (IgGOB+, 26 patients) had higher intrathecal IgG indexes (*p* < 0.01), lower values of BAFF Index (11.9 ± 6.1 vs 17.5 ± 5.2, *p* < 0.01), and higher CSF CXCL13 levels (27.7 ± 33.5 vs 0.9 ± 1.5, *p* < 0.005). In these patients, BAFF Index but not CSF CXCL13 levels inversely correlated with the intrathecal IgG synthesis (*r* > 0.5 and *p* < 0.05 for all correlations). CSF leukocyte counts were significantly higher in IgGOB+ compared to IgGOB− (*p* < 0.05) and HC (*p* < 0.01), and correlated to CSF CXCL13 concentrations (*r* 0.77, *p* < 0.001).

The gCTh was significantly lower in patients with higher CSF CXCL13 levels (2.41 ± 0.1 vs 2.49 ± 0.1 mm, *p* < 0.05), while no difference in MRI parameters of WM and GM pathology was observed between IgGOB+ and IgGOB−.

**Conclusions:**

The intrathecal IgG synthesis inversely correlated with BAFF Index and showed no correlation with CSF CXCL13. These findings seem to indicate that intrathecally synthesized IgG are produced by long-term PCs that have entered the CNS from the peripheral blood, rather than produced by PCs developed in the meningeal follicle-like structures (FLS). In this study, CXCL13 identifies a subgroup of MS patients characterized by higher leukocyte counts in the CSF and early evidence of cortical thinning, further suggesting a role for this chemokine as a possible marker of disease severity.

## Background

Several lines of evidence suggest that B cells play a central role in the pathogenesis and/or the clinical evolution of multiple sclerosis (MS). First, it is well known that the great majority of MS patients have quantitative (increased indexes) and/or qualitative (presence of IgG oligoclonal bands, IgGOB) immunoglobulin changes in the cerebrospinal fluid (CSF), indicating an intrathecal Ig production [1, 2]. Second, histological studies have disclosed the presence of complement deposition and plasma cells (PCs) in white matter (WM) lesions [3]. Third, B cell follicle-like structures (FLS) are observed in the meninges of patients with progressive MS [4] and correlate with the degree of meningeal inflammation and cortical demyelination, and with a higher rate of disability progression [5]. Finally, the treatment of MS patients with anti-CD20 monoclonal antibodies determines a significant reduction in the number of both active brain lesions and in clinical relapses [6–9].

The role of B cells in MS pathology may be multifaceted [7]. Beside autoantibody production [10, 11], B cells may present central nervous system (CNS) antigens with co-stimulatory signals leading to activation and proliferation of effector T cells, may produce pro-inflammatory cytokines (e.g. IL-6, IL-12, and TNF), or may regulate the immune response by providing IL-10, a cytokine that suppresses the T cell functions either directly or indirectly [12] (i.e., differentiation of T regulatory cells and the suppression of dendritic cell function [7, 10–12]).

The presence of meningeal follicle-like structures (FLS) has stimulated the hypothesis of a “germinal center reaction” within the CNS of MS. Briefly, naïve B cells recruited in the CNS (throughout CXCL13 signal) may locally meet and present CNS antigens to T helper lymphocytes. Both B and T cells actively collaborate (respectively as centroblast and follicular T helper, that produce IL-21, the “signature” of an ongoing follicular reaction) to establish a germinal center reaction that leads B cell maturation to IgG-secreting PCs. PCs will survive within the CNS thanks to intrathecally produced B cell survival factors, such as B cell-activating factor (BAFF), as suggested by a recent observation [13]. On the other hand, we cannot exclude that FLS formation may be a mere epiphenomenon of MS, expression of a chronic meningeal inflammation, not directly involved in intrathecal Ig synthesis.

We studied serum and CSF levels of soluble factors involved in naïve B cell recruitment (CXCL13), in follicular reaction (IL-21) and intrathecal B cell survival (BAFF) in MS patients at clinical onset and correlated their intrathecal synthesis with local Ig production and with magnetic resonance imaging (MRI) parameters of white and gray matter damage. The aim of the study was to explore whether, at clinical onset, B cell-related cytokines could serve as immunological markers of MS severity/prognosis.

## Methods

### Patients

Forty consecutive patients with a diagnosis of clinically isolated syndrome suggestive of MS (CIS) or early-onset relapsing-remitting MS (eRRMS), whose diagnosis was achieved in agreement with the most recent diagnostic criteria [14], were enrolled in the study. Inclusion criteria were as follows: (i) age between 18 and 50 years, (ii) no medical history of steroid therapy in the previous 30 days, (iii) no medical history of immunomodulating or immunosuppressive therapy, and (iv) no gadolinium-enhancing lesion at the MRI performed at the study entry.

Seventeen healthy controls (HC) were also enrolled in the study. These subjects complained tension headache, subjective transient sensory symptoms, psychosomatic disorders, etc. that underwent lumbar puncture for diagnostic purposes having unexpected, subtle, but unspecific white matter lesions at MRI. Immunological and coagulation screenings, microbiological and standard CSF analysis were normal; also upper aortic artery and Transcranic ultrasound EcoColorDoppler and visual evoked potentials were normal.

No differences for age and gender were observed between eRRMS and HC (Table 1). The study was approved by the local ethics committee and a written informed consent was obtained by all the participants.

**Table 1:** Standard serum and CSF parameters in HC and CIS/eRRMS

	HC	CIS/eRRMS
Gender (F/M)	13/4	26/14
Age at LP (years)	43.2 ± 9.2	37.8 ± 10.0
Disease duration (years)	n.a.	0.5 ± 0.9
CSF [Alb] (mg/dL)	17.0 ± 5.1	22.5 ± 9.8
Serum [Alb] (mg/dL)	4370.6 ± 379.0	4260.0 ± 333.6
Q_Alb_ (10^−3^)	3.9 ± 1.2	5.3 ± 2.3*
CSF [IgG] (mg/dL)	2.2 ± 0.9	3.7 ± 1.8***
Serum [IgG] (mg/dL)	1141.2 ± 215.2	1042.0 ± 197.7
Q_IgG_ (10^−3^)	1.9 ± 0.6	3.6 ± 1.7****
IgG Index	0.5 ± 0.1	0.7 ± 0.3***
IgG Loc (mg/dL)	0.1 ± 0.3	5.9 ± 10.2*
IgIF (%)	0 ± 0	11 ± 17*
IgGOB (%)	0%	65%****
Leukocyte counts (/μL)	2.1 ± 1.1	6.9 ± 7.7*

Disease duration was defined as the time between clinical disease onset and lumbar puncture. For gander, IgIF% and IgGOB chi-square test was applied. For all the other variables, a *t* test was performed

*HC* healthy controls, *CIS/eRRMS* CIS suggestive of MS or early RRMS*, LP* lumbar puncture, *n.a.* not applicable, *Csf* cerebrospinal fluid, *S* serum, *IgGOB* IgG oligoclonal bands

*p < 0.05; **p < 0.01; ***p < 0.005; ****p < 0.001

### CSF and serum routine analysis

CSF was collected by non-traumatic lumbar puncture between 8:00 and 9:00 am. Routine examination on paired CSF and serum specimens included cell count and differentiation, CSF/serum IgG ratio (Q_IgG_), CSF/serum albumin ratio (Q_Alb_) to estimate the integrity of the blood-brain barrier (BBB), calculation of intrathecal IgG synthesis by means of quantitative formulae (IgG Index) [2] IgG Reiber’s Hyperbolic Function for IgG intrathecal synthesis fraction (IgGIF) [1] and Local Production (IgGLoc) [1], and demonstration of IgGOB by isoelectric-focusing and specific IgG immunofixation. BBB damage was considered when Q_Alb_ was higher than the normal value for patient’s age (i.e., age/15 + 4). After cell centrifugation, both CSF and serum were stored at −80 °C until cytokine analysis.

### Cytokine determination

CSF and serum levels of BAFF (R&D System, Cat. Numb. DBLYS0B), IL-21 (Bender MedSystems Cat. Numb. BMS2043TEN), and CXCL13 (R&D System, Cat. Numb. DCX130) were measured by a highly sensitive immune-enzymatic assay ELISA according to the manufacturer’s instruction. BAFF concentrations (pg/mL) were measured on 1:2 diluted sera and on native CSF. IL-21 and CXCL13 concentrations (pg/mL) were measured on native sera and CSF. Cytokine ratio (CSF/serum cytokine: Q_BAFF_, Q_CXCL13_, and Q_IL-21_) and Index (CSF/serum cytokine/CSF/serum albumin: BAFF Index, CXCL13 Index, IL21 Index) were calculated.

### MRI protocol

Images were acquired using a 3T scanner (Ingenia, Philips Medical Systems, Best, The Netherlands) with 33 mT/m power gradient and a 32-channel head coil. No major hardware upgrades occurred during the study, and bimonthly quality-assurance sessions assured measurement stability. The following images were acquired for each subject: (a) three-dimensional (3D) turbo field echo (TFE, 3D-T1): repetition time (RT) 7.8 ms; echo time (ET) 3.6 ms; 180 contiguous axial slices with the off-center positioned on zero with thickness of 1.0 mm; flip angle = 8°; matrix size = 220 × 220; FOV = 220 × 220 × 180 mm^3^. This sequence was acquired before and after gadolinium administration. (b) 3D-fluid attenuated inversion recovery (FLAIR): RT 4800 ms; ET 310 ms; inversion time (IT) 1650 ms; 365 contiguous axial slices with thickness of 1.0 mm; matrix size 256 × 256; and FOV = 256 × 256 × 182 mm3; (c) 3D-double inversion recovery (DIR): RT 13,000 ms, ET 10 ms, IT 3400/325 ms; 40 contiguous axial slices, resolution 1 × 1 × 3 mm, FOV 230 × 200 mm, time 3.5 min. Two experienced observers, blinded to the patient’s identity, assessed all images. The global cortical thickness (gCTh) was analyzed by means of Freesurfer on 3D-T1 sequences. WM lesions were identified on FLAIR sequences, while cortical lesions (CL) were identified on DIR scans by two blinded evaluators (PD, PS) using published consensus recommendations.

### Statistical analysis

For normally distributed variables, the *T* test (when two groups were compared) or the ANOVA (for more than two group comparisons) were performed applying the Bonferroni correction. For ordinal categorical variables, the Mann-Whitney *U* test was performed, while for no ordinal variables, Pearson’s chi-square test was used. Linear correlation between variables was tested using Pearson’s single or multiple linear model when all variables were normally distributed. The significance level was set at *p* < 0.05.

## Results

### Intrathecal IgG synthesis in HC and CIS/eRRMS

Table 1 summarizes routine serum and CSF findings in HC and CIS/eRRMS. As expected, all the indexes of intrathecal IgG synthesis (IgG CSF, Q_IgG_, IgG Index, IgGLoc, and IgGIF) were significantly higher in CIS/eRRMS compared to HC. Higher Q_Alb_ values were observed in CIS/eRRMS (5.3 ± 2.3 × 10^−3^) than in HC (3.9 ± 1.2 × 10^−3^, *p* < 0.05), but only six CIS/eRRMS patients (15 vs 0% in HC, *p* = 0.2) had BBB damage.

### Intrathecal cytokine synthesis in HC and CIS/eRRMS

#### CXCL13

In HC, CXCL13 was found in all sera and, at very low levels (0.9 ± 1.5 pg/mL), in 8/17 (47%) CSF. In these subjects, no correlation was observed between Q_CXCL13_ and Q_Alb_ (*r* 0.2, *p* = 0.4), even when only CXCL13-positive CSF were included in the analysis (*r* 0.7, *p* = 0.1). Since these data indicated that CXCL13 was intrathecally produced, rather than derived from passive filtration from blood, only the CSF concentrations of this cytokine were considered in further analyses. CXCL13 was detected more frequently (75%, *p* < 0.05) and at higher concentration (19.7 ± 29.3 pg/mL, *p* < 0.05) in the CSF of CIS/eRRMS than HC.

#### IL-21

In HC, IL-21 was detectable in all CSF (28.1 ± 29.1 pg/mL) but only in one serum, thus suggesting that CSF IL-21 was intrathecally produced. No difference in CSF IL-21 levels was observed between HC (28.1 ± 29.1 pg/mL) and CIS/eRRMS (20.7 ± 22.8 pg/mL, *p* = 0.3).

### BAFF

BAFF was detected in all the sera and CSF of HC and CIS/eRRMS. In HC Q_BAFF_ mildly correlated to Q_Alb_ (*r* 0.4), indicating that CSF BAFF may result from both choroid plexus filtration and active production within the CNS. The BAFF Index was lower in CIS/eRRMS (12.4 ± 5.5 pg/mL) than in HC (17.5 ± 5.2 pg/mL, *p* < 0.005).

### IgGOB-based stratification of CIS/eRRMS

**Fig. 1:**
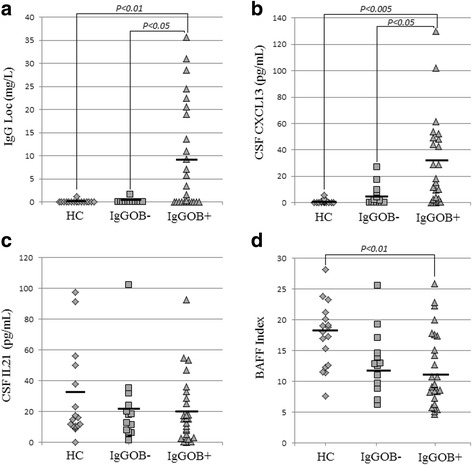
IgG and cytokine concentrations in the CSF. IgG Loc expressed in mg/dL (**a**), CSF CXCL13 and IL-21 concentrations (pg/mL; **b**, **c**), and BAFF Index values (**d**) in HC and CIS/eRRMS with (IgGOB+) or without (IgGOB−) intrathecal synthesis of IgG oligoclonal bands. IgGOB+ patients had increased CXCL13, normal IL-21, and decreased BAFF Index compared to HC and IgGOB− patients. Only significant comparison is indicated. *Abbreviations: HC* healthy controls, *CIS/eRRMS* CIS suggestive of MS or early RRMS, *LP* lumbar puncture, *n.a*. not applicable, *CSF* cerebrospinal fluid, *S* serum. *IgGOB+*: patients with IgG oligoclonal bands in the CSF; *IgGOB−*: patients without IgG oligoclonal bands in the CSF

Based on the presence of IgGOB in the CSF, CIS/eRRMS patients were divided in IgGOB positive (IgGOB+, 26 patients) and in IgGOB negative (IgGOB−, 14 patients) (Table 2). The analysis based on this stratification disclosed that all the differences previously found between HC and CIS/eRRMS were confirmed for IgGOB+ patients, but not for IgGOB− patients (Fig. 1a). Furthermore, serum IgG concentrations in IgGOB− were lower than in HC (*p* < 0.01) and IgGOB+ (*p* < 0.05).

**Table 2:** Serum and CSF parameters in HC, IgGOB−, and IgGOB+ patients

	HC	IgGOB−	IgGOB+
Gender (F/M)	13/4	9/5	17/9
Age at LP (years)	43.2 ± 9.2	37.2 ± 10.7	38.2 ± 9.7
Disease duration (years)	n.a.	0.3 ± 0.6	0.6 ± 1.1
Csf-[Alb] (mg/dL)	17.0 ± 5.1	21.2 ± 8.7	23.1 ± 10.4
S-[Alb] (mg/dL)	4370.6 ± 379.0	4225.0 ± 297.7	4278.8 ± 355.6
Q_Alb_ (10^−3^)	3.9 ± 1.2	5.0 ± 2.0	5.4 ± 2.4*
Csf-[IgG] (mg/dL)	2.2 ± 0.9	2.4 ± 0.8	4.5 ± 1.8**** °°°°
S-[IgG] (mg/dL)	1141.2 ± 215.2	944.6 ± 124.2**	1094.3 ± 211.6°
Q_IgG_ (10^−3^)	1.9 ± 0.6	2.6 ± 1.0	4.2 ± 1.8**** °°
IgG Index	0.5 ± 0.1	0.5 ± 0.1	0.8 ± 0.3**** °°°
IgG Loc (mg/dL)	0.1 ± 0.3	0.2 ± 0.6	9.0 ± 11.5** °
IgIF (%)	0 ± 0	1.1 ± 2.7	16.4 ± 18.5*** °°
Leukocyte counts (/μL)	2.1 ± 1.1	2.9 ± 2.4	9.0 ± 8.6*** °
Csf -[BAFF] (pg/mL)	67.3 ± 21.6	69.9 ± 27.3	50.7 ± 20.5* °
BAFF Index	17.5 ± 5.2	13.2 ± 5.1	11.9 ± 6.1**
Csf-[CXCL13] (pg/mL)	0.9 ± 1.5	5.0 ± 8.2	27.7 ± 33.5*** °
Csf-[IL21] (pg/mL)	28.1 ± 29.1	21.6 ± 25.3	20.2 ± 21.9

IgGOB+ patients differed from both BOIgG− and HC for intrathecal IgG synthesis parameters, CSF CXCL13 concentrations, and BAFF Index values. For gender, IgIF%, and IgGOB, chi square test was applied. For all the other variables, a *t* test was performed. Abbreviations as in Table 1

*P* values compared to HC: *p < 0.5; **p < 0.01; ***p < 0.005; ****p < 0.001; *p* values compared to IgGOB−: °*p* < 0.5; °°*p* < 0.01; °°°*p* < 0.005; °°°°*p* < 0.001

CSF CXCL13 concentrations were higher in IgGOB+ compared to both IgGOB− (*p* < 0.05) and HC (*p* < 0.005) (Fig. 1b). A higher number of IgGOB+ patients had detectable CXCL13 in the CSF (77%) compared to IgGOB− (50%), but the difference was not significant (*p* = 0.2).

No difference in CSF IL-21 levels was observed between HC, IgGOB−, and IgGOB+ (Fig. 1c).

As expected on the base of previous results, CSF BAFF levels and Index were significantly lower in IgGOB+ (50.7 ± 20.5 pg/ml, *p* < 0.05, and 11.9 ± 6.1, both *p* < 0.005, respectively) but not in IgGOB− (69.9 ± 27.3 pg/ml and 13.2 ± 5.1, respectively, *p* = 0.8 and *p* = 0.1) compared to HC (Fig. 1d).

### Correlation analysis

**Fig. 2:**
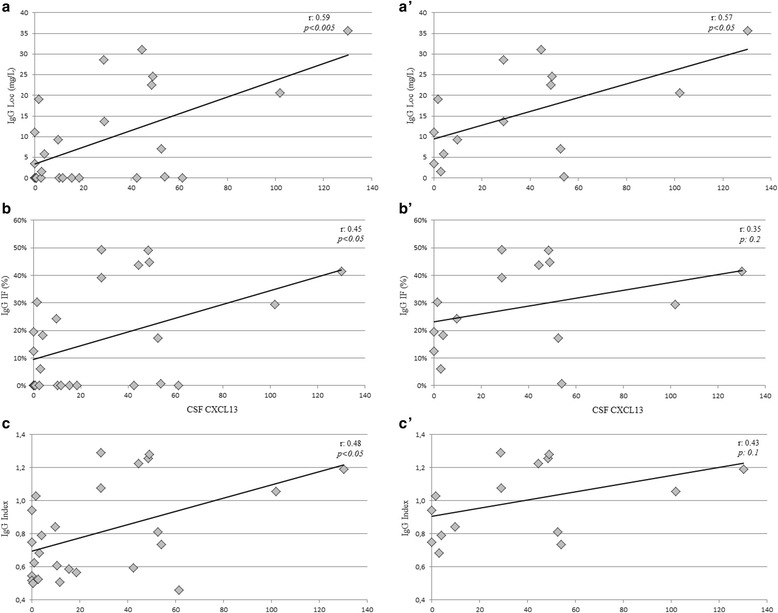
Correlations between CSF CXCL13 and intrathecal IgG synthesis. A mild correlation was observed between CSFCXCL13 and intrathecal IgG synthesis when all the patients were included in the analysis (**a**–**c**). When the null values for intrathecal IgG synthesis were excluded, the correlation was weak (**a’**) or lost (**b’**, **c’**)

**Fig. 3:**
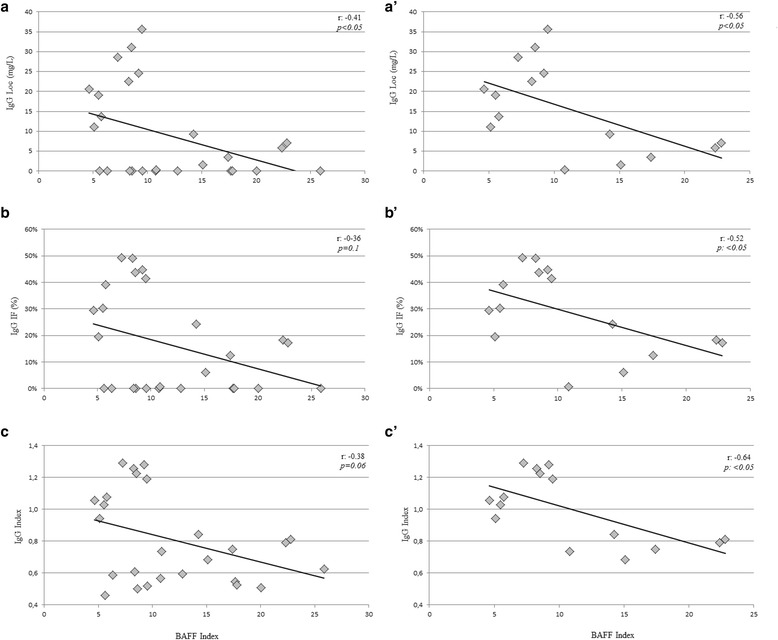
Correlations between BAFF Index and intrathecal IgG synthesis. A mild correlation between BAFF Index and intrathecal IgG synthesis was observed when all the patients were considered (**a**–**c**). The correlation was strong when the null values of intrathecal IgG synthesis were excluded (**a’**–**c’**)

For the correlation analysis, only quantitative continuous variables, namely IgG Index, IgGLoc, and IgGIF, were included as dependent variables. Table 3 shows the correlation between the parameters of intrathecal IgG synthesis and BAFF Index or CSF CXCL13 concentrations in IgGOB+. CSF CXCL13 concentrations correlated with all the parameters of intrathecal IgG synthesis (Fig. 2a–c), while BAFF Index mildly correlated only with IgGLoc values (Fig. 3a–c). In order to avoid the influence of null values on the direct correlation and considering that IgGOB+ presented null values for CSF CXCL13, we focused on patients with quantitatively increased intrathecal IgG synthesis. In 15 IgGOB+ patients having IgG Index, IgGLoc, and IgGIF values above the normal range of reference, only BAFF Index correlated to all these parameters (Fig. 3a’–c’), while CXCL13 concentrations correlated only with IgGLoc (Fig. 2a’).

**Table 3:** Correlation between intrathecal IgG synthesis parameters and BAFF Index or CXCL13

(A) All patients	BAFF Index	
IgG Loc (mg/dL)	−0.41*	0.59***
IgIF (%)	−0.36	0.45*
IgG Index	−0.38	0.48*
(B) Only IS+	BAFF Index	LCS-CXCL13
IgG Loc (mg/dL)	−0.56*	0.57*
IgIF (%)	−0.52*	0.35
IgG Index	−0.64*	0.43

The correlation was calculating on overall population of BOIgG+ patients (A) or including only patients with quantitatively increased intrathecal IgG synthesis (IS+, B). *R* values derived from Pearson’s single linear model are represented. Other abbreviations as in Table 2

*p < 0.5; **p < 0.01; ***p < 0.005

Finally, BAFF Index did not correlate to CSF CXCL13 concentrations in HC (*r* −0.36, *p* = 0.2), MS patients (*r* −0.29, *p* = 0.1), IgGOB− (*r* −0.05, *p* = 0.9), and IgGOB+ (*r* −0.31, *p* = 0.1).

### CSF leukocyte counts in CIS/eRRMS and in IgGOB+ and IgGOB−

CSF leukocyte number was significantly higher in CIS/eRRMS (6.9 ± 7.7/μL) compared to HC (2.1 ± 1.1 μL, *p* < 0.05). CSF CXCL13 concentrations, but not BAFF Index, significantly correlated to leukocyte counts (*r* 0.77, *p* < 0.001, Table 4).

**Table 4:** Leukocyte counts in CIS/eRRMS correlates to CSF-CXCL13

	*r*	*r* ^2^	*p* value
CIS/eRRMS			
CSF-[CXCL13] (pg/mL)	0.79	63.2%	<0.001
BAFF Index	−0.21	4.4%	0.2
IgGOB+			
CSF-[CXCL13] (pg/mL)	0.77	59.3%	<0.001
BAFF Index	−0.22	4.7%	0.3
IgGOB−			
Csf-[CXCL13] (pg/mL)	0.34	11.6%	0.3
BAFF Index	0.22	4.8%	0.5

Subgroup analysis revealed that this correlation was confirmed only in IgGOB+ patients. Abbreviations as in Table 2. *R* values derived from Pearson’s single linear model are represented

BOIgG+ had higher CSF leukocyte number (9.0 ± 8.6/μL) than IgGOB− (2.9 ± 2.4/μL, *p* < 0.05) and HC (*p* < 0.01). In IgGOB+, CSF CXCL13 concentrations, but not BAFF Index values, significantly correlated to leukocyte number (*r* 0.77, *p* < 0.001, Table 4), while no correlation between leukocyte number and all the other CSF parameters was observed in BOIgG−.

### MRI parameters in CIS/eRRMS

No difference in gCTh was observed between HC and CIS/eRRMS. However, a higher variability in gCTh was found in CIS/eRRMS (range 2.25–2.67 mm) compared to HC (2.34–2.58 mm). In order to find associations between CSF (IgGOB detection, BAFF Index, CSF CXCL13, IgG Index, IgGLoc, IgG IF) and MRI parameters, we performed a subgroup analysis.

On the base CXCL13 concentrations in the CSF of HC, we found a cutoff value of 6.8 pg/mL (*μ* + 4*δ*) that allowed the identification of two groups of CIS/eRRMS patients having CSF CXCL13 values below (CXCL13−, 20 patients) or above (CXCL13+, 20 patients) this limit. All HC had CXCL13 values below this limit.

BAFF Index was not applicable in this analysis, since its range did not differ between the two groups and therefore a cut-off value was meaningless.

In as much as the three quantitative indexes of intrathecal IgG synthesis gave highly concordant values, only IgG Loc was considered for further analysis (values ≤0.0 = IgGLoc−; values >0.0 = IgGLoc+).

**Fig. 4:**
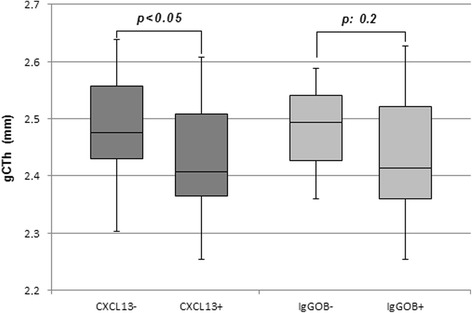
Global cortical thickness is reduced in patients with higher CSF CXCL13. MS patients with higher CSF CXCL13 (CXCL13+) concentrations presented a significant cortical thinning compared to patients with lower values (CXCL13−)

Interestingly, no difference in any MRI parameter was observed between IgGOB+ and IgGOB− or between IgGLoc− and IgGLoc+. CXCL13+ patients had a significant thinning of gCTh (2.42 ± 0.09 mm) compared to CXCL13− (2.48 ± 0.10 mm, *p* < 0.05; Fig. 4). The results did not change when a different cut-off value (*μ* + 3*δ*, i.e., 5.2 pg/mL) was applied.

No association was found between any CSF parameters and WM or GM lesion number and volume.

## Discussion

Intrathecally produced IgGOB can be demonstrated in the great majority of MS patients at clinical onset, persist in the CSF throughout the patient’s life, and constitute the major evidence of B lymphocytes and PC involvement in MS pathology [3]. The origin of long-term secreting PCs, likely the source of intrathecally synthesized IgG, has not been established yet. Moreover, the progressive colonization of MS meninges by FLS suggests that local B cell maturation to PCs may support the intrathecal synthesis of IgM and IgG [5]. However, since FLS accumulation seems to parallel disease progression (i.e., they were demonstrated in high percentage in progressive MS cases, rarely in RRMS and not at clinical onset), we would expect to observe qualitative modifications of the IgGOB patterns over the course of the disease. On the contrary, longitudinal studies demonstrated that IgGOB are almost qualitatively stable [15–19].

B cells have several other functions than producing antibodies [12]. They may act as efficient antigen-presenting or cytokine-secreting cells, thus enhancing T cell response. Therefore, the pathogenic role of B cells in the FLS may be multifaceted. FLS have been observed in association with cortical inflammation, gray matter demyelination, and various degrees of microglia activation [4]. Moreover, meningeal and cortical inflammation were found to correlate with some clinical parameters, such as median age at disease onset, time to disease progression, time to wheelchair dependence, and age at death [4]. Thus, the analysis of intrathecally synthesized B cell-related cytokines/chemokines may give the opportunity to define the timing of B cell recruitment and FLS formation in the CNS and to explore the role of FLS in the pathogenesis and clinical progression of MS.

In a previous study [13], we observed that BAFF Index correlated to quantitative parameters of intrathecal IgG synthesis in MS at clinical onset and suggested a BAFF-dependent PCs survival within the CNS. In this study, we analyzed the intrathecal production of cytokines involved in B cell recruitment and follicular maturation in order to explore the origin of intracerebral PCs.

CXCL13, a chemokine that is not physiologically produced within the CNS, was increased in the CSF of CIS/eRRMS, especially in IgGOB+ patients. This finding suggests that CXCL13, produced by activated microgial cells, becomes detectable since the early disease phases, and is responsible for the recruitment of naïve B cells within the CNS. On the other hand, BAFF, constitutively produced in the CNS by astrocytes, is absorbed by local antibody-producing PCs, thus explaining the decreased levels of this cytokine in early MS stages. The previously described increased BAFF levels in the CSF of progressive MS [20] might found an explanation in the more diffuse astroglial proliferation and in the decreased burden of inflammation that characterize the more advanced phase of MS. However, since CSF CXCL13 was detected, although rarely, in patients with no evidence of intrathecal IgG synthesis, is not associated with BAFF Index and correlates only mildly with IgGLoc, we may argue that the majority of naïve B cells recruited in the CNS do not undergo the process of maturation to PCs. Taken all together, these findings do not speak in favor of a germinal center reaction within FLS, at least in early MS phases, and are in line with the qualitative stability of IgGOB patterns during the course of MS.

An interesting finding of our study was the association of CSF CXCL13, but not BAFF Index, with cortical thinning since early MS phases. This observation seems to link a soluble factor that plays a pivotal role in the B cell recruitment in the CNS, to a MRI parameter of neurodegeneration (gray matter atrophy), that is correlated to disability progression in MS.

Our findings are in line with the previous reports on the association of CXCL13 to leukocyte CSF counts [20], higher risk of relapse, and CIS conversion to MS [21], as well as with higher disease activity in the WM [22, 23], further suggesting a possible role of CXCL13 as a soluble marker of disease activity or severity in MS.

Other cytokines were found to increase in MS CSF [24]. Among these, the macrophage/microglia-derived interleukin-1 beta (IL-1β) was also found to correlate with cortical thinning [25]. The finding that both IL-1β and CXCL13 can be found in the CSF of very early MS and both factors associate with cortical thinning further suggests a pivotal role of microglia in MS pathology. Indeed, in vitro, CXCL13 is produced by monocytes, and at much higher levels by macrophages, and CXCL13 mRNA and protein expression is induced by TNFα and IL-1β [26]. All these data suggest that activated and proliferating microglia cells (a characteristic histological feature of cortical pathology) produce large amounts cytokines, such as IL-1β and CXCL13, that attract and maintain T and B cells in the inflamed meninges and in the cortex of MS.

## Conclusions

Our findings indicate that intrathecally synthesized IgG are produced by PCs that enter the CNS from the peripheral blood at a given time and are locally retained as long-term PCs. A role for FLS as source of intrathecal IgG in early disease phases is not supported by our observations on CXCL13.

In our study, CXCL13 identifies a subgroup of MS patients characterized by the early evidence of cortical thinning, further suggesting a role for this chemokine as marker of disease severity.

## Abbreviations

BAFF: B cell-activating factor
BBB: Blood brain barrier
CIS: Clinical isolated syndrome
CL: Cortical lesions
CNS: Central nervous system
CSF: Cerebrospinal fluid
DIR: Double inversion recovery
eRRMS: Early-onset relapsing-remitting multiple sclerosis
FLAIR: Fluid attenuated inversion recovery
HC: Healthy control
IgGIF: IgG intrathecal fraction
IgGLoc: IgG locally produced
IgGOB: IgG oligoclonal bands
MS: Multiple sclerosis
PCs: Plasma cells
Q_Alb_: CSF/serum albumin
Q_BAFF_: CSF/serum BAFF
Q_CXCL13_: CSF/serum CXCL13
Q_IgG_: CSF/serum IgG ratio
Q_IL-21_: CSF/serum IL-21
WM: White matter
